# Three-dimensional mass spectrometry imaging (3D MSI): incorporating top-hat IR-MALDESI and automatic *z*-axis correction

**DOI:** 10.1007/s00216-025-05755-w

**Published:** 2025-02-03

**Authors:** Alexandria L. Sohn, John G. Witherspoon, Robert C. Smart, David C. Muddiman

**Affiliations:** 1 FTMS Laboratory for Human Health Research, Department of Chemistry, North Carolina State University, Raleigh, NC 27695 USA; 2 Department of Biological Sciences, North Carolina State University, Raleigh, NC 27695 USA; 3 Center for Human Health and the Environment, North Carolina State University, Raleigh, NC 27695 USA

**Keywords:** 3D, Mass spectrometry imaging, Lipids, Automatic *z*-axis correction, Top-hat

## Abstract

Leveraging a depth profiling approach expands the chemical elucidation of mass spectrometry imaging techniques to another dimension. Three-dimensional MSI (3D MSI) reveals the distribution of analytes with greater anatomical detail to add another level of information in a biological study. Infrared matrix-assisted laser desorption electrospray ionization (IR-MALDESI) has demonstrated utility for an ablation-based approach, enabling simplified sample preparation workflows and streamlined data processing pipelines compared to a serial-sectioning strategy. To improve 3D MSI on the IR-MALDESI platform, two technologies have been characterized in tandem for the intention of minimizing sampling bias: (1) a top-hat optical train and (2) a chromatic confocal probe (CA probe). While the modified optical train creates a square spot size to avoid a Gaussian ablation crater after the analysis of subsequent layers, the CA probe enables automatic *z*-axis correction (AzC) to maintain the laser’s focus on the surface of the sample. The work herein demonstrates the integration and optimization of these technologies on mouse skin, motivated by the clear biological skin layers that result in differential lipid expression and subsequent detection. Results support that a laser energy of 1.3 mJ/burst with the top-hat optical train and a 120 µm step size in the X and Y dimensions presented a comparable depth resolution to previous studies at under 7 µm. Further, the optimized parameters were utilized on two biological replicates to evaluate method reproducibility where lipid annotations and their abundance were considered.

**Supplementary Information:**

The online version contains supplementary material available at 10.1007/s00216-025-05755-w.

## Introduction

Mass spectrometry imaging (MSI) offers insights into spatial biology, reporting hundreds to thousands of analytes in a sample in a label-free manner. Diverse sample types have been used [1–4] where intact tissue sections are often implemented to correlate histology with analyte detection to inform biomedical research and other relevant fields [5]. While context is provided in the *x*- and *y*-dimensions in conventional MSI analyses, biology is a three-dimensional (3D) phenomenon that requires interrogation from all angles to be fully understood. Some of the most common 3D imaging modalities are medical diagnostic tools, such as magnetic resonance imaging (MRI) and computerized tomography (CT), and are used to highlight abnormal anatomical structures [6, 7]. Complementing these results with a mass spectrometric perspective can offer powerful insights to the underlying biology of a disease or condition; the inherent dimensionality of biology drives the necessity for developing other 3D approaches to provide a comprehensive understanding of a system of interest. Therefore, the informativeness of MSI can be extended beyond a 2D tissue with 3D MSI, where chemical signatures can also be associated with morphological features in the *z*-dimension.

Recently, several MSI platforms have demonstrated 3D imaging to enhance our understanding of material science and biological systems, where different platforms offer certain features and advantages [1]. For instance, secondary ion mass spectrometry (SIMS) employs a primary ion beam for a surface sputtering, or depth profiling, approach where sample material is continuously analyzed at nm to µm increments until achieving the desired sample depth [1]; with this technique, the elemental composition of materials and single cells has been elucidated [8, 9] along with drugs of interest and target fragment ions in mouse ear tissue [10]. Desorption electrospray ionization (DESI) and matrix-assisted laser/desorption ionization (MALDI) are better suited for biological tissue samples, as they offer softer ionization to retain intact biomolecules [11]. However, due to the nature of surface sampling for both of these techniques and intolerance for deviations in sample topography, a serial-sectioning approach is necessary for 3D MSI by DESI [12] or MALDI [13, 14]. This constitutes cryo-sectioning 2D serial sections throughout the entire specimen of interest, performing the relevant sample preparation, individually imaging each tissue, and then reconstructing the tissue sections for downstream data analysis [11]. This workflow is extensive and requires precision at all phases during the execution of the experiment, leaving room for analytical variability in numerous capacities [11]. Further, because the tissues are not being imaged through the complete thickness of the tissue, some tissue remains unanalyzed and results in a loss of biological information between each section. Finally, the coregistration and reconstruction of the images are not trivial and necessitate sophisticated software tools for accurate tissue alignment and proper biological interpretation [15].

In an ablation-based sampling technique, instead of imaging numerous 2D serial sections, a tissue of interest can be imaged repeatedly in technical layers across the same region of interest (ROI). This largely mitigates the challenges affiliated with serial-sectioning, as the tissue remains intact, is prepared a single time for analysis, and does not require complex image reconstruction. Beyond this, since the same ROI is imaged repeatedly, no tissue is left unanalyzed along with its respective biological context. This approach has been demonstrated by several mid-infrared laser systems (e.g., 2970 nm), including both laser ablation electrospray ionization (LAESI) [16, 17] and laser ablation remote atmospheric pressure photoionization/chemical ionization (LARAPPI/CI) [18], both for the purposes of studying small molecules in plants. More extensively, infrared matrix-assisted laser desorption electrospray ionization (IR-MALDESI) has demonstrated its feasibility on both hard and soft sample types, such as pharmaceutical pills [19, 20] and mouse skin [21]. While all of these platforms have similar features and overcome challenges of throughput and robustness associated with a serial-sectioning approach, sampling biases are introduced with a Gaussian laser profile and from tackling changes in sample topography.

A Gaussian laser profile is common with laser-based platforms, where the energy of the beam is highest at the center and decreases exponentially along the edges to produce circular spot shapes [22]. Depth profiling propagates the central energy bias of a Gaussian beam throughout each technical layer, resulting in a Gaussian-shaped ablation crater [22]. Additionally, since an ablation-based approach does not involve sectioning a tissue into flat sections, the topography across the sample surface is subject to change, especially in the first technical layer. This results in the sample moving out of the focal plane of the laser, ultimately rendering inconsistent laser ablation between voxels that would support inaccurate biological conclusions [20, 23].

Recent work on the IR-MALDESI platform has focused on technology to address both of these concerns in a 3D MSI experiment. First, a custom diffractive optical element (DOE) [18, 22] was introduced to the laser’s optical train to homogenize the energy distribution. This promotes a reproducible, square ablation pattern that, in turn, produces a top-hat crater profile during ablation-based 3D MSI [22]. With respect to topography changes across the surface of a sample, Xi et al. have integrated a chromatic confocal probe (CA probe) into the IR-MALDESI source for automatic *z*-axis correction (AzC) [20]. Before MS imaging, the sample is scanned by the CA probe to evaluate deviations from the laser’s focal plane and record the necessary adjustments in the *z*-dimension. The CA probe data are automatically transferred to the source control software [24] to guide voxel-by-voxel AzC, ensuring reproducible ablation across the entire ROI [20].

Despite the development of these innovations, their integration and optimization for the purposes of 3D IR-MALDESI-MSI have yet to be characterized. This motivates the work herein, where we utilized top-hat IR-MALDESI-MSI with AzC for 3D experiments on mouse skin. As part of the aims of this study, we optimized the *z*-resolution for this workflow to resolve lipid species to the anatomical layers of the skin, specifically the epidermis. The heterogeneity of lipid detection was resolved through the depth of the tissue, and the optimized protocol was demonstrated on two other biological replicates to evaluate the reproducibility of this work and advise improvements for future studies.

## Experimental

### Sample preparation

All aspects of animal care and experimentation described in this study were conducted according to the NIH guidelines and were approved by the NC State University Institutional Animal Care and Use Committee (IACUC). Mouse livers were provided by the Ke Cheng Lab in the College of Veterinary Medicine (IACUC Protocol #19–811-B) and were fresh frozen after harvesting. Until sectioning and analysis, the liver tissue was maintained in a − 80 °C freezer. For evaluating laser spot dimensions, tissue sections were cut with a cryostat (Leica CM 1950, Buffalo Grove, IL, USA) at − 15 °C and 7-µm thickness [25] on the same day of analysis.

The mouse skin in this work was prepared according to a previous report by Bai et al. [21] and was used for both method development and reproducibility studies. The Smart Lab in the Department of Biological Sciences (IACUC Protocol 23–219) provided SKH1 hairless mouse skin for analysis. Within 3–5 min of sacrifice by cervical dislocation, the dorsal skin was excised and cut into 6 square sections of tissue approximately 1 × 1 cm in size. The tissue sections were thaw-mounted on aluminum foil before being placed in a pre-cooled weigh boat to flash-freeze over an ethanol (190 proof, VWR International, King of Prussia, PA, USA) and dry ice bath. Samples were kept in the bath for 15 min and then secured onto a pre-frozen glass slide (VWR International, Portsmouth, NH, USA) before being placed on dry ice. The skin samples were maintained at − 80 °C in a freezer until the time of analysis.

### Incorporating the top-hat optical train and chromatic confocal sensor system on the IR-MALDESI platform

Experiments on liver tissue and mouse skin were both performed on the NextGen IR-MALDESI platform, which has been described in detail in previous reports [26]. In short, the NextGen source positions an electrospray (ESI) emitter in-line with the ion transfer tube of the mass spectrometer (MS), where the sample of interest rests below on a three-dimensional translational stage. Many matrices have been utilized for experiments on this platform [27–30]; however, no exogenous matrix is used in the analyses reported herein [21, 22]. Once an ROI is defined on the sample, the stage will move in defined step sizes and a laser will fire at each discrete XYZ coordinate to ablate neutral species from the sample. The sample material will interact with the charged droplets from the ESI plume above the stage to promote ionization, enabling the mass spectrometer to readily detect the respective species at each coordinate location [31].

When the top-hat IR-MALDESI [22] and automatic *z*-axis correction [20] were combined, a modified laser optical train and chromatic confocal probe sensor (CA probe) (Precitec GmbH & Co. KG, Gaggenau, Germany) were respectively incorporated into the source. First, the mid-IR laser (2970 nm, JGM Associates, Inc., Burlington, MA, USA) was fired at a rate of 10 Hz [32] through an optical train containing a beam expander and collimator (CaF_2_, *f* =  − 75 mm and + 250 mm, Thorlabs, Newton, NJ, USA), which were separated by 175 mm. To produce a square spot size, the Gaussian beam was homogenized and transformed by a custom diffractive optical element (DOE, FO-1933, #2, HOLO-OR) [22] before being focused on the sample with an aspheric lens (CaF_2_, *f* =  + 25 mm, Thorlabs). Burn paper (ZAP-IT, Edmund Optics, Barrington, NJ, USA) was used to confirm that the laser was in focus and to aid in determining the coordinate position of where the laser was firing. This step preceded calibration of the CA probe to ensure corroboration between topographical measurements and *z*-axis correction during MSI analysis [20]. Coregistration of these positions was imperative for the IR-MALDESI source to accurately determine the sample topography across the ROI, then subsequently correlate the appropriate *z*-axis adjustments during imaging experiments. This workflow and further details regarding the communication between source components are extensively described by Xi et al [20].

### Three-dimensional top-hat IR-MALDESI-MSI with automatic z-axis correction on skin

Method development experiments were conducted only in positive mode with an ESI solvent composition of 50% acetonitrile (Fisher Scientific, Hampton, NH, USA) in LC–MS-grade water (Fisher Scientific) with 0.2% formic acid modifier (99.5 + %, Sigma-Aldrich, Carlsbad, CA, USA). In the reproducibility studies that followed, the optimized method was conducted in both positive and negative modes for broader lipid coverage. The negative mode solvent was 50% methanol (Fisher Scientific) in LC–MS-grade water with 1 mM acetic acid (Fisher Scientific).

To stabilize the ESI plume, a high voltage was applied (3.0–4.0 kV) with an optimized flow rate (1.0–1.5 µL/min). As mentioned, no exogenous matrix was applied to the sample for analysis, and the tissue was maintained at ambient conditions during the experiment. The IR-MALDESI source is coupled to an Orbitrap Exploris™ 240 (Thermo Fisher Scientific, Bremen, Germany), and data were collected in centroid mode to reduce data file size and improve the speed of downstream processing [33]. The EASY-IC internal standard (fluoranthene, [M^•+^] *m/z* 202.0777) was activated during analysis for high mass accuracy measurements, and the automatic gain control was disabled with a fixed injection time of 15 ms [34]. A resolving power (RP) of 240,000_FWHM_ at (*m/z* 200) was used for all runs, and the mass range of interest was *m/z* 200–1000.

**Fig. 1:**
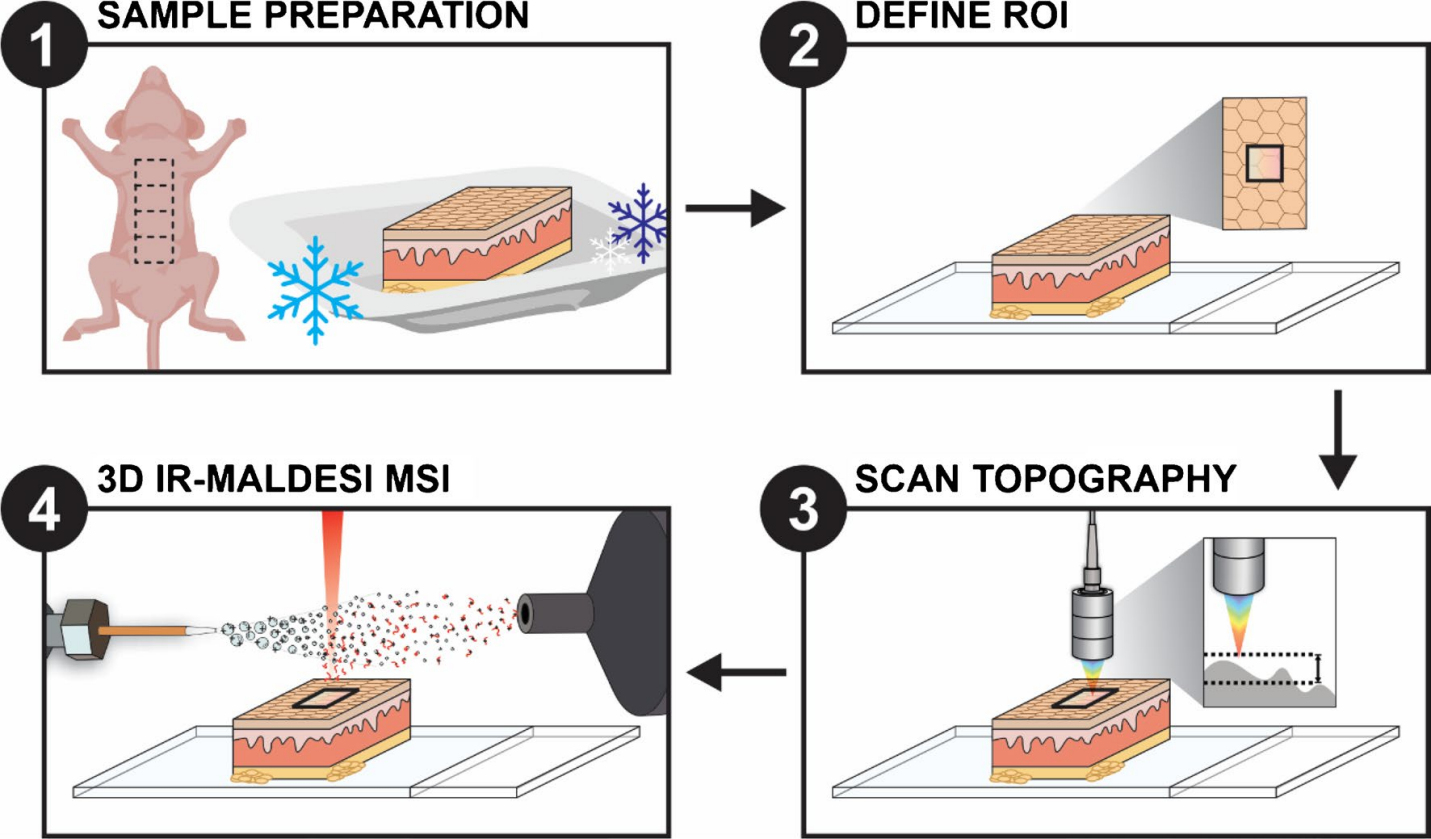
A summary of the experimental workflow for 3D MSI by top-hat IR-MALDESI with the incorporation of the CA probe for automatic *z*-axis adjustment. For MSI analysis, the defined ROI is scanned under the CA probe for topography measurements to inform AzC during imaging. Steps 3 and 4 are repeated for the desired number of technical layers

Once the relevant optical train and CA probe was incorporated and calibrated within the IR-MALDESI source, RastirZ was used to specify the ROI and record topography measurements from the CA probe. When the experimental run is initiated, the sample was first rastired beneath the CA probe to detect and record deviations in the z-axis that require adjustments during the imaging experiments.  After this was completed, the sample was translated to laser position, and the MSI analysis ensued. At each respective XY coordinate, the sample stage is adjusted appropriately in the *z*-dimension to maintain the focus of the laser. This workflow was repeated for the desired number of layers for 3D MSI and is summarized in Fig. 1.

During the method development work, various laser energies were tested (i.e., 1.0–2.1 mJ/burst) and their spot sizes were confirmed on mouse liver tissue, which are reported in Supplemental Figure S1. Once 3D MSI was pursued, all ROIs were imaged from the same mouse specimen, and step sizes of 110, 120, and 130 µm (both *x*- and *y*-dimensions) were evaluated. ROIs were 5 × 5 voxels for a total of 25 scans per technical layer, and *z*-resolution determination required imaging 5, 10, 15, and 20 layers in separate experiments. Once the optimized parameters were defined, the workflow was repeated on two biological replicates of the same genotype which was confirmed by PCR genotyping of genomic DNA (data not shown). Depth profiling ROIs were 10 × 10 voxels in size and all experiments analyzed 10 technical layers into the tissue. To estimate the ablation depth per layer for reproducibility studies, the total depth at the end of the analysis was divided by the number of layers imaged (*n* = 10).

### Data analysis

After staining mouse liver tissue with Histogene and preserving in Permount mounting medium (Fisher Scientific, Nazareth, PA, USA), a Leica LMD 7000 microscope (Leica Biosystems) was used to collect optical images and measure laser spot dimensions on liver tissue. Mouse skin after 3D MSI was measured by a confocal laser scanning microscope (VK-X1100, Keyence, Itasca, IL, USA) to determine the depth resolution and obtain measurements of the crater profiles. These data were analyzed in MultiFile Analyzer (Keyence).

For correlation of histological features, an adjacent section of skin tissue was preserved from one of the replicate mice. SKH1 hairless mice (8–12 weeks of age) were euthanized and whole dorsal skin was laid flat on card stock, fixed in 10% phosphate-buffered formalin, and then transferred to 70% ethanol after 24 h. Tissues were embedded in paraffin using standard processing settings and then sectioned at 5-µm and placed onto charged slides. Slides were stained with routine hematoxylin and eosin (H&E) stains. Morphometric microscopic analysis was done using Nikon NIS Elements.

All data were converted from.RAW to mzML by MSConvert, before subsequent conversion to imzML format with imzML converter [35]. Files were uploaded to METASPACE annotation software and tentative identifications were provided using the LipidMaps Database (LMDB) at a false discovery rate (FDR) of 10% [36]. Other data processing, visualization, and removal of background ions were performed in MSiReader Pro v2.60 (MSI Software Solutions, LLC, Raleigh, NC, USA) [37, 38]. All tentative lipid identifications provided in this work are supported by high mass measurement accuracy (± 2.5 ppm) and spectral accuracy.

Ion images shown are representative of a single technical layer in the analysis, as if from a top-down or aerial view. When showing ion images with all layers from this perspective, the technical layers are numbered accordingly (Fig. 5B). Alternatively, ROIs were converted into line scan images in order of analysis and stitched together to provide a side profile of the 3D experiment (Fig. 5C). 3D heat map plots were generated, where individual ions were maintained at the same abundance scaling. Additionally, these ions were viewed as 3D colocalization plots and a single ion occupied each channel (i.e., red, green, blue) to visualize overlap in detection with respect to abundance, as appropriate (Fig. 6).

Where appropriate, the associated SMART metrics [39] are reported with ion images from MSI analyses, which clearly state critical parameters and results for ease of interpretation. The “S” metric reports the step size and total number of scans collected, “M” is the molecular identification confidence (i.e., MS1) and the MMA window used for the image, “A” is the number of annotations where applicable, “R” is the resolving power used during experimentation, and “T” is the time of acquisition for the experiment [39]. For the last metric in particular, this will pertain to the time per technical layer imaged.

## Results and discussion

This work demonstrates the incorporation of a DOE for top-hat MSI and CA probe for AzC in 3D MSI to optimize a robust workflow for 3D MSI of mouse skin and inform work on other model systems in the future. While these technologies have been characterized individually, they have not been utilized together and optimized for 3D MSI. The lipid signatures within the biological layers of mouse skin are distinct and were analyzed for this work, as this model has been studied on the IR-MALDESI platform previously [21]. The laser energy and step size were optimized to obtain the best *z*-resolution of these skin layers prior to deeper biological investigations.

### Method optimization and z-resolution determination

One of the major aims of this work was to minimize the amount of tissue ablated per technical layer and render the best *z*-resolution possible while maintaining adequate signal abundance. Therefore, it was critical to utilize a laser energy that promotes square spot sizes and results in sufficient ion signal from the sample while enabling as many technical layers as possible through the depth of the tissue. Step size was also a consideration because overlapping, or oversampling, of voxels results in more tissue being ablated for each technical layer and must be avoided to improve *z*-resolution.

When utilizing the DOE in the optical train, a higher energy applied from the laser is beneficial in retaining the square spot shape due to the homogenization of laser energy; however, this is counterintuitive to minimizing the ablation volume. Previous energy optimizations are not informative to this work, as a different optical train was utilized on the platform [21] and a different focusing element [22] has been considered here for improved spatial resolution. Therefore, prior to 3D MSI experiments, laser energies were tested from 1.0 to 2.1 mJ on sectioned mouse liver tissue (7-µm thickness [25]) and the spot sizes were measured, which are reported in Supplemental Figure S1. This range was tested as square spots were consistently observed from 1.0 mJ and higher. Additionally, the ratio of the *x* and *y* measurements of the spot dimensions were calculated. When applying a laser energy of 1.3 mJ/burst, the spot size was consistently observed around 128 µm × 128 µm with a dimension ratio of 1.00. This supported continuing method optimization further with this laser energy for the optical train of interest.

Next, step sizes of 110 µm, 120 µm, and 130 µm in both the *x*- and *y*-dimensions were tested on mouse skin to evaluate the respective crater profile and the *z*-resolution. Before imaging analyses, CA probe data was collected to inform AzC for mass spectrometry analyses.

**Fig. 2:**
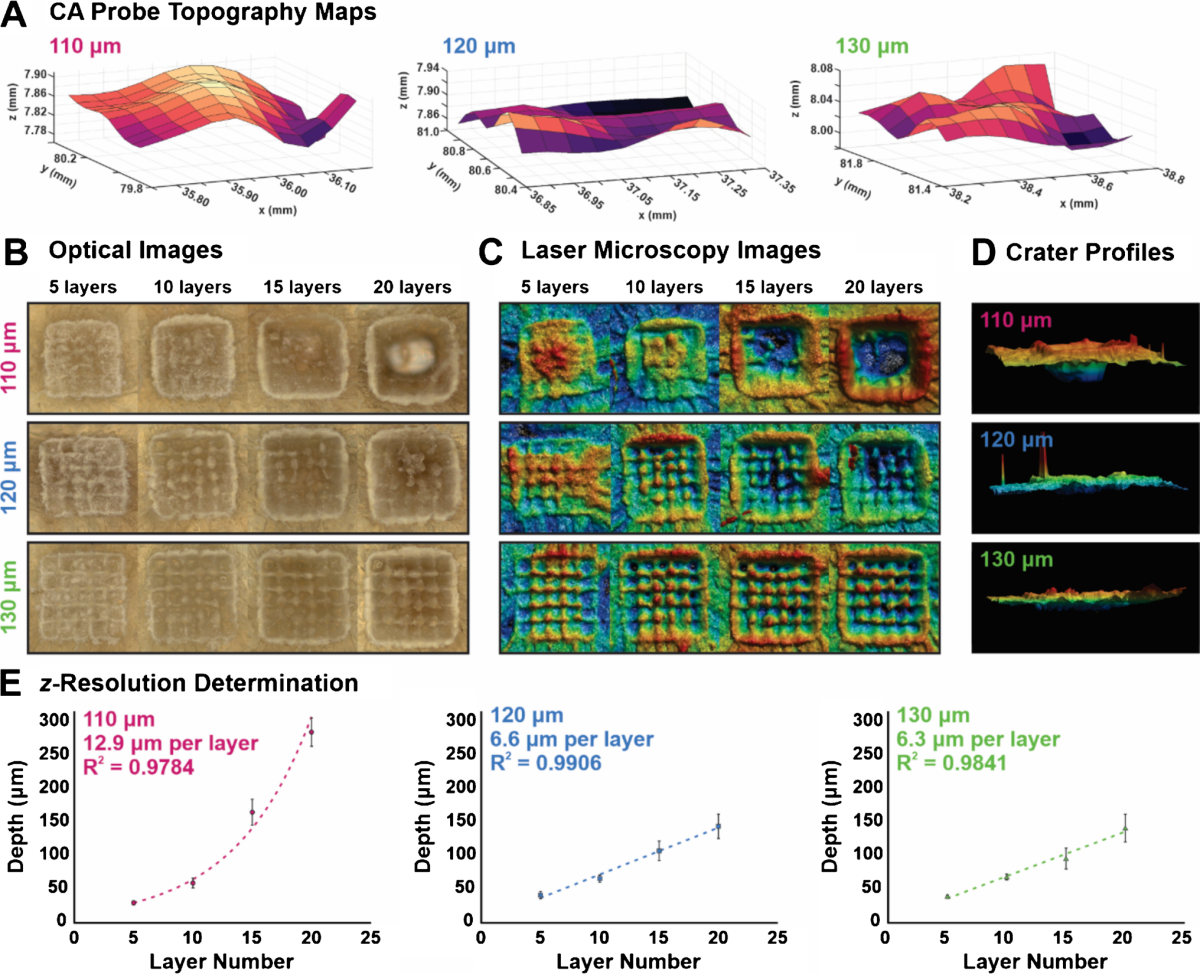
Using a laser energy of 1.3 mJ/burst, three step sizes were evaluated (110, 120, and 130 µm) with the aims of minimizing the amount of sample ablated for each layer to optimize the *z*-resolution in imaging experiments. **A** CA probe data show sample topography, where the *z*-axis was corrected at each voxel to maintain the laser’s focus. **B** Optical images and **C** laser microscopy images show an aerial view of the ROIs used for *z*-resolution determination, along with **D** the crater profiles from the side of the ROI at 20 technical layers. **E** The number of technical layers was plotted against the ablation depth and fit to a curve for *z*-resolution determination

Figure 2A shows topography plots collected prior to each step size test, demonstrating the ability for the technology to adapt to any unique surface structure for reproducible imaging, which is particularly critical for a laser-based imaging platform. Each of the three step sizes were imaged with 5, 10, 15, and 20 technical layers to observe the crater morphology and determine the *z*-resolution for each condition (Fig. 2B–D). Optical and laser microscopy images of the craters from an aerial view (Fig. 2B and C) show complete ablation of the ROI with a step size of 110 µm, where the entire tissue was imaged through at 20 layers. With step sizes of 120 µm and 130 µm, residual tissue between voxels in the *x*- and *y*-dimensions leave peak-like artifacts, and this was more pronounced with the 130-µm step size. This characteristic was likely attributed to the residual heating energy along the sides of the laser profile; even though there was some oversampling with the step size of 120 µm; for example, the energy along the sides remains lower than in the center and causes lack of ablation towards the edges of the ablation spot. Spots were undersampled with a step size of 130 µm, meaning that lack of tissue imaging between voxels also attributes to the peak artifacts in the crater. The side profiles of the ablation craters reflect the observations from the aerial perspective (Fig. 2D), where utilizing the smallest step size of 110 µm preserved the top-hat crater, but 120 µm and 130 µm did not ablate through the tissue completely at 20 layers.

The ablation craters were evaluated more quantitatively by measuring the ablation depth at the various number of technical layers analyzed. At 5, 10, 15, and 20 layers under each step size condition, depth measurements were taken and plotted to calculate the *z*-resolution with the correlation coefficient reported (Fig. 2E). While both 120 µm and 130 µm showed a linear correlation between the number of technical layers and the ablation depth, this relationship was exponential for the 110 µm step size condition. In this case, despite the top-hat crater being maintained with the smallest step size, the *z*-resolution of approximately 13 µm is inferior to the other parameters and the exponential relationship between ablation depth and layer quantity is suboptimal. Alternatively, the other two step sizes tested resulted in an average ablation depth just less than 7 µm; this *z*-resolution is similar to results produced with a Gaussian beam from previous work [21] but avoids sampling bias towards the center of an ROI. Even though the depth resolution was comparable between 120 µm and 130 µm step sizes, this showed support for 120 µm to be selected as the optimal parameter since less undersampling, and therefore less non-analyzed tissue, was observed at this laser energy.

**Fig. 3:**
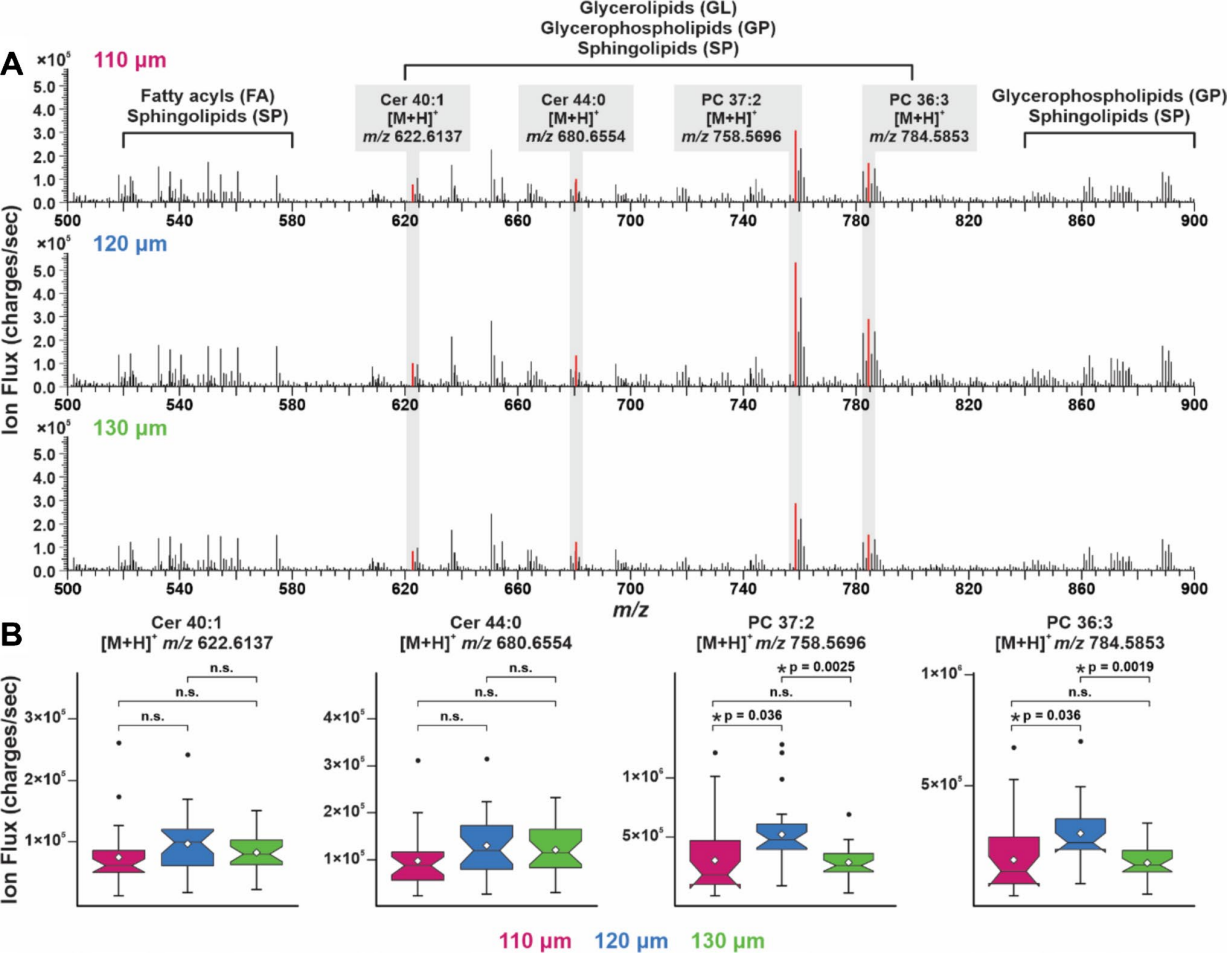
The step sizes of interest were further interrogated to evaluate spectral differences in the first technical layer of analysis. **A** The mass spectra were similar between conditions, where various lipid categories (e.g., fatty acyls, glycerophospholipids) were detected and four tentatively identified lipids were compared at each step size**. B** The signal of these ions was compared and tested for significance

MS data were also consulted to support the ideal parameters for this work, where data from the first technical layer were used such that the same biological region of the tissue was being analyzed. When comparing the mass spectra of each step size tested in Fig. 3A, the signal of sample-related peaks appeared comparable and showed detection of various lipid species from several categories (e.g., glycerophospholipids, sphingolipids). More specifically, four tentatively identified lipids were highlighted in the mass spectra and their ion flux was compared with each step size in notch box plots (Fig. 3B), including Cer 40:1 (*m/z* 622.6137), Cer 44:0 (*m/z* 680.6554), PC 37:2 (*m/z* 758.5696), and PC 36:3 (*m/z* 784.5853); these data show the ion flux detected for all the scans in the first technical layer of the skin and are color coordinated to correspond to the respective step size. When their ion flux was evaluated with respect to step size, 120 µm showed a trend of higher signal than the other two step sizes. While the statistical significance of this trend is dependent on lipid species, the observation that signal was improved at a step size of 120 µm demonstrates more support that this parameter was optimal for the aims herein.

### Detecting lipid heterogeneity

**Fig. 4:**
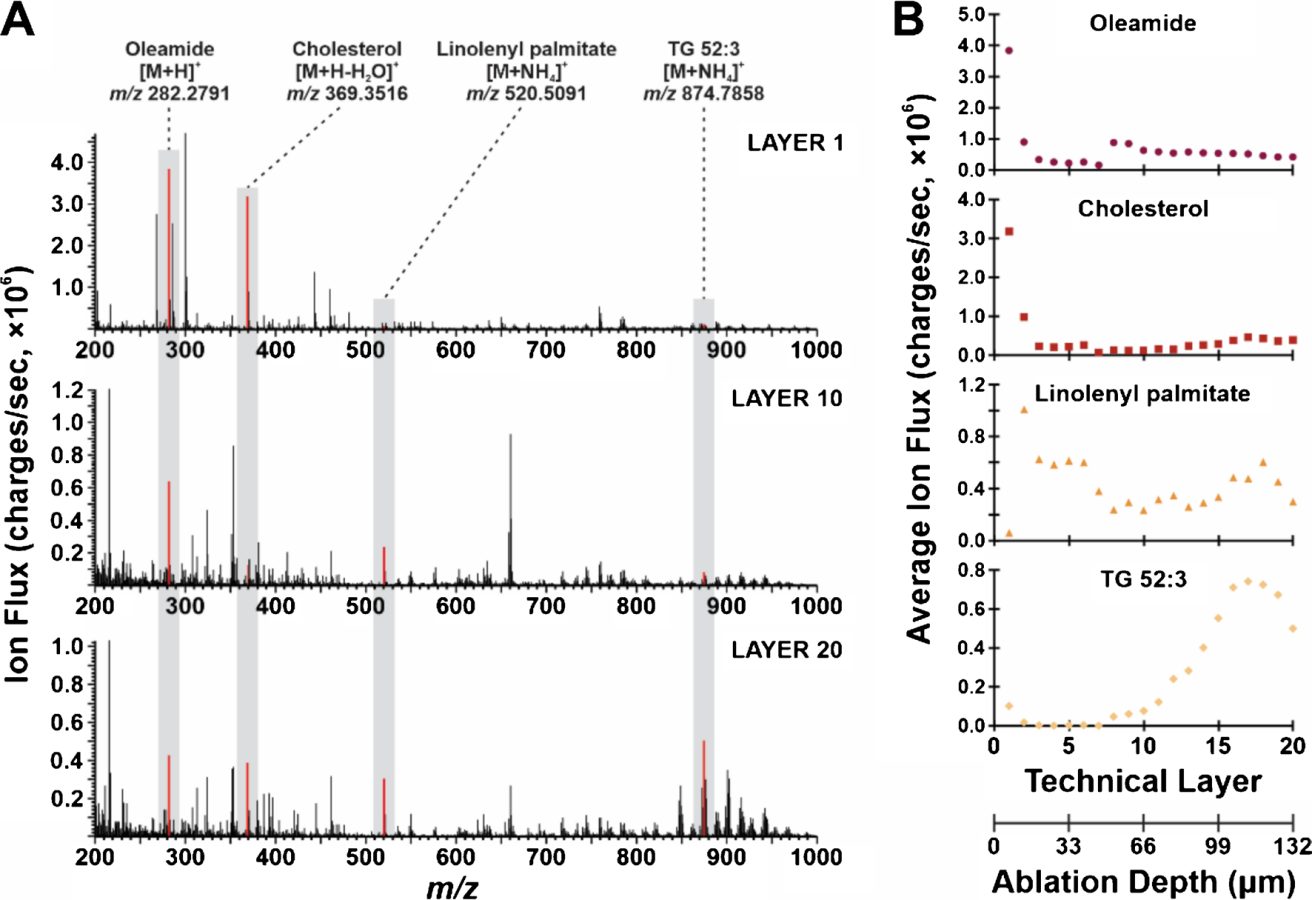
As the depth of the tissue is sampled, lipid heterogeneity is observed. **A** At optimized sampling conditions (1.3 mJ/burst, 120 µm step size), technical layers 1, 10, and 20 show different features in their mass spectra (average of 25 scans in ROI). Four tentatively identified lipids are highlighted (i.e., oleamide, cholesterol, linolenyl palmitate, TG 52:3) as their abundances change in the depth of the tissue. **B** The average abundance of the four species was plotted against the respective technical layer. The ablation per layer was estimated as 6.6 µm, enabling the correlation of ablation depth to the number of technical layers imaged

Lipid heterogeneity was investigated across 20 layers using optimized parameters. Figure 4A shows the mass spectra at different technical layers, revealing stark differences in the detection of lipid species as the anatomy of the tissue changes. The most apparent difference is the increase in lipid detection from *m/z* 700–1000 as the depth profiling continues through the tissue, but four specific analytes were highlighted and analyzed throughout the mass range of interest. For example, tentatively identified oleamide (*m/z* 282.2791) is much higher in abundance in technical layer 1 than subsequent layers, and this was reflected in the plot shown in Fig. 4B; as the tissue is imaged deeper, oleamide’s signal varies but trends downward and cholesterol (*m/z* 369.3516) follows a similar pattern. Conversely, linolenyl palmitate (*m/z* 520.5091) and TG 52:3 (*m/z* 874.7858) respectively vary at different technical layers and trend upward as we reach deeper ablation depths. These lipids indicated biological changes in the system that likely correspond to anatomical features.

**Fig. 5:**
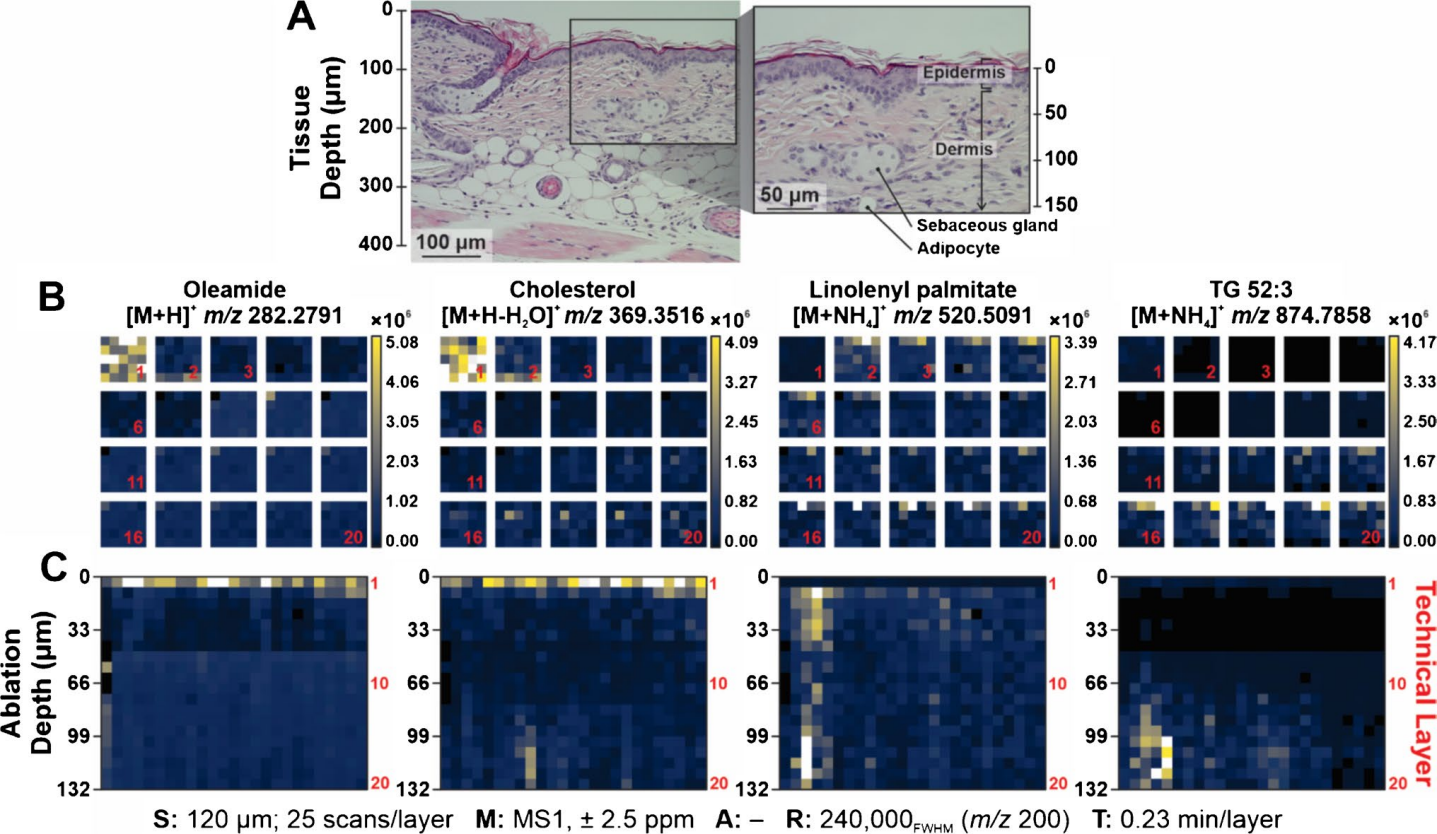
**A** Labeled histology of hairless SKH1 mouse skin, including two major skin layers (i.e., epidermis, dermis) and some anatomical features. The larger image was taken at 20 × magnification, and the callout box is at 40 × magnification. **B** Ion images can be visualized from an aerial perspective, where technical layers are shown in order starting from the top left and guided by the red numbers. **C** Alternatively, individual ROIs can be viewed as a line and stitched together to better visualize changes as the depth of the tissue is imaged. The red numbers on the right side of each ion image correlate to the technical layer, while the ablation depth is indicated on the left side of each image. The SMART information for this experiment is included beneath the images

Adjacent skin was H&E stained and annotated, as provided in Fig. 5A, where the epidermis, dermis, and a sebaceous gland are labeled. Ion images for each technical layer are shown for oleamide, cholesterol, linolenyl palmitate, and TG 52:3, where the red numbers in Fig. 5B guide what layer each of the images is from. An alternative visualization is shown in Fig. 5C, where each ROI is depicted as a row in order of analysis, where each row sequentially shows ion abundance as a function of depth. As Fig. 4 previously plotted the change of signal during the 3D MSI analysis, this is now visualized for each ion through the depth of the tissue. Additionally, the respective SMART metrics are included here to concisely describe essential information pertaining to data collection and results, such as the step size, annotation confidence, and resolving power [39].

Aligning with previous observations, both cholesterol and oleamide are most abundant in the first technical layer of the analysis which corresponds to the epidermal layer of the tissue. This corroborates existing reports in literature, as fatty acids and cholesterol are critical species in the barrier function of the stratum corneum, which is the outermost layer of the epidermal skin layers [40, 41]. Additionally, the signal of linolenyl palmitate and TG 52:3 are localized to certain regions of the ROI in the dermis of the skin. When evaluating the line scan heatmaps (Fig. 5C), it can be noted that both of these lipids congregate in a bulb-like structure in a similar shape to a sebaceous gland that was ablated on the top edge of the ROI. While taking on a similar morphology, literature supports that the detection of these species is likely pronounced in sebaceous glands [42–44]. Therefore, not only does this technology enable the resolution of different biological skin layers, but also demonstrates that anatomical features can be resolved by chemical localization alongside histology.

**Fig. 6:**
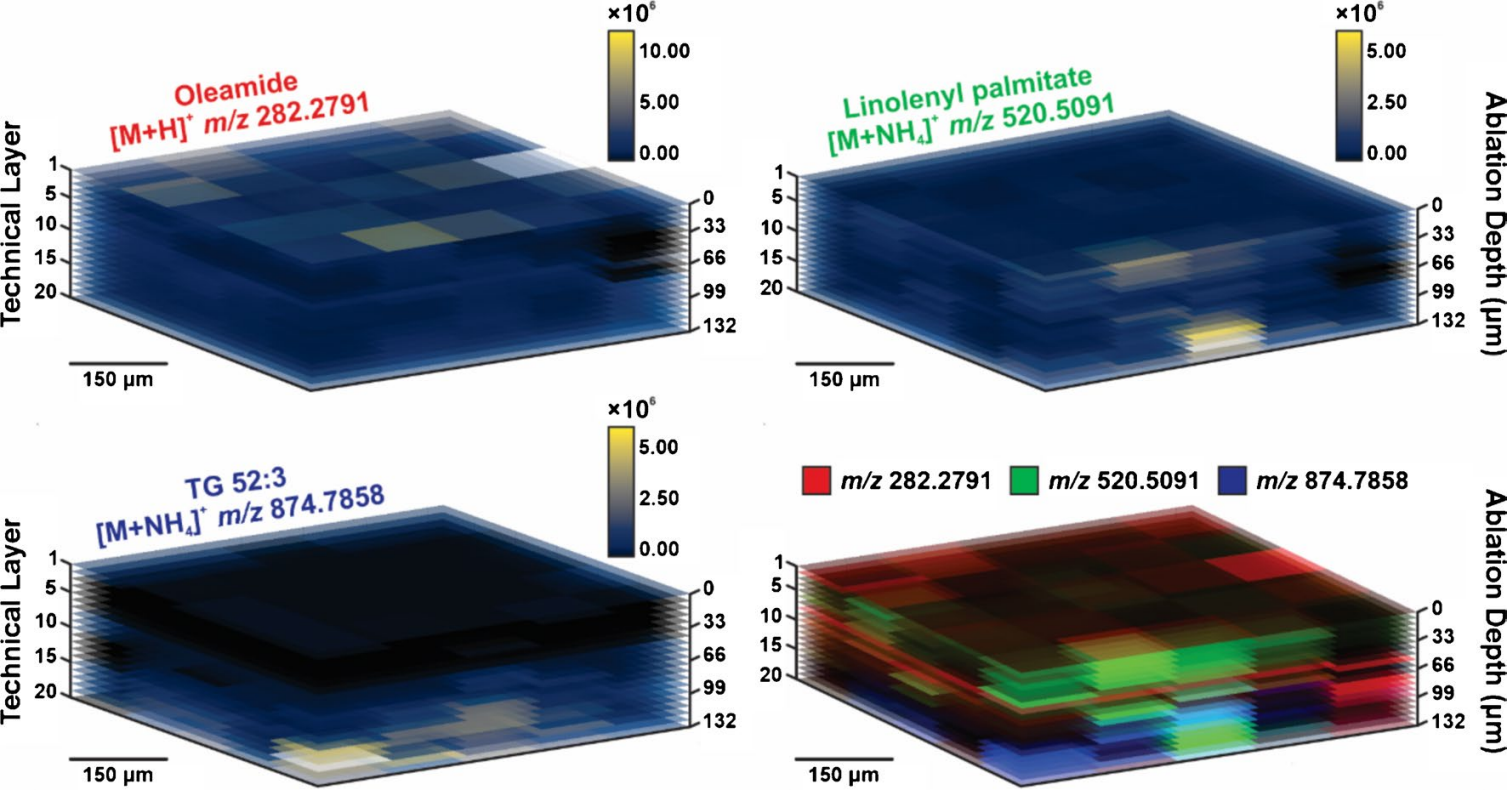
3D ion images and colocalization plots show lipid heterogeneity throughout the skin. Each technical layer shows differences in detection for oleamide, linolenyl palmitate, and TG 52:3 throughout the depth of the tissue, where technical layers and their ablation depths are shown. When these ions are colocalized together as different colors (bottom right) their overlap and dominance in signal can be visualized and correlated with tissue anatomy

The same localization was also observed in 3D heatmaps, as shown in Fig. 6. Three of the species of interest that have been discussed to this point are shown individually or can each be represented as a single color to highlight colocalization. In the colocalization plot, the red channel is occupied by oleamide where this species is largely abundant in the first technical layer of the tissue. Consistent with previous discussion, TG 52:3 and linolenyl palmitate were more abundant in deeper layers of the tissue and localize to a particular region of the depth profile. This is evident in the colocalization plot as well, since the blue and green channels follow a similar pattern of accumulation in the ROIs. The information shown in this visualization is similar to the ion images shown previously but offer another perspective of analysis that can be conducted for 3D MSI studies to interrogate biology.

### Evaluating method reproducibility

**Fig. 7:**
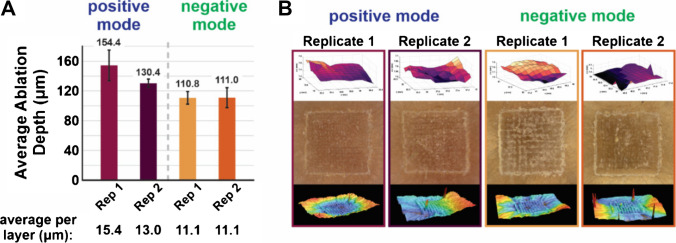
**A** The ablation depths during reproducibility studies varied from the original optimization work. These studies included two biological replicates in positive and negative mode, where 10 technical layers were imaged with ROI dimensions of 10 × 10 voxels with the optimized parameters. The average *z*-resolutions are reported based on 10 measurements and were based on the average total crater depth after analyzing 10 technical layers. **B** CA probe plots from the first technical layer of each analysis are provided. Optical images of the tissue from an aerial perspective are provided with profiles of the ablation craters. The sharp peaks in the crater profiles (bottom row) are an artifact of residual hair from the mouse skin that is out of the range of the measurement for laser microscopy

To investigate the method’s reproducibility, two biological replicates were analyzed in both positive and negative mode to evaluate the consistency of ablation depth, crater morphology, and lipid annotation consistency. The crater profiles were analyzed similarly to the method development work, and the respective optical and laser microscopy data are reported in Fig. 7.

The average *z*-resolution varied from method development, demonstrating a range of 11.1–15.4 µm being ablated for each technical layer. The increase in ablation depth with these experiments should not be overlooked, particularly if the goal of a study is to optimize the *z*-resolution of a sample. However, it can be noted that these results still improve from a higher laser energy used with a Gaussian beam [21] while also providing the advantages of correcting for sample topography and more homogenous sampling through the ROI. This is seen in the optical images and crater profiles in Fig. 7B, as there are still peak-like structures in between voxels in the analysis but the ablation crater is not excessively sampled towards the center of the ROI. Such observations are consistent with the analyses conducted during method development experiments.

**Fig. 8:**
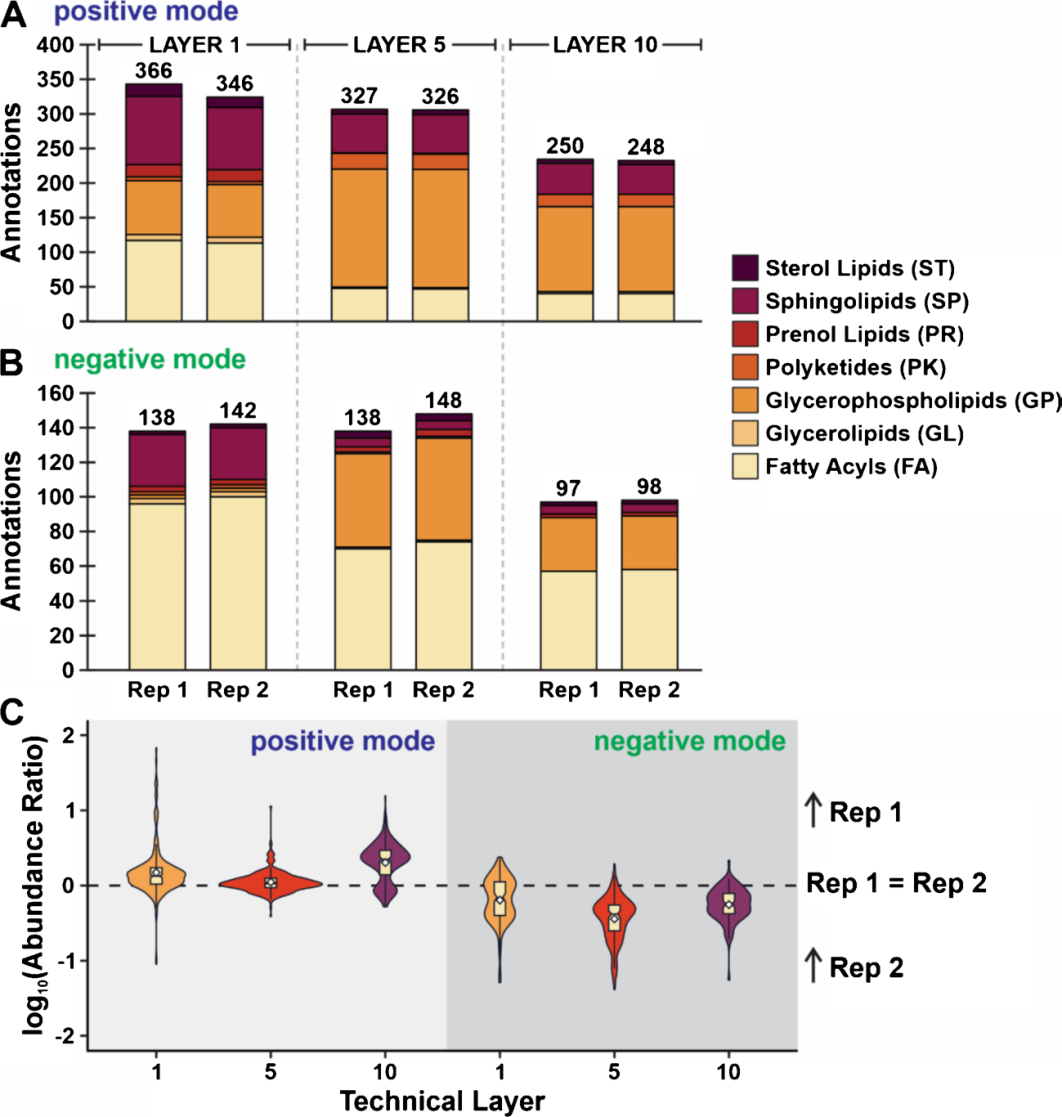
Lipid annotations are reported for both **A** positive and **B** negative mode, where the respective lipid categories are color coded. **C** For all common annotations, the detected ion abundance was averaged for each replicate. The ratio of the average abundances (Replicate 1:Replicate 2) was calculated and converted to a log_10_-scale to evaluate method reproducibility. If the abundance of a lipid was the same for both replicates at a certain layer, the log_10_(abundance ratio) is equal to zero. If this value was positive or negative, this indicates higher average abundance in Replicate 1 or 2, respectively. Violin plots were utilized to show the distribution of the data. Notch box plots are also included to show the 95% confidence interval of the median, and white diamonds indicate the mean of the values

Finally, lipid annotations and their ratio of abundances between the biological replicates were considered to support method reproducibility. Species were annotated in technical layers 1, 5, and 10 and are differentiated by their lipid category in Fig. 8A and B for positive and negative mode, respectively. Results suggest strong agreement in the lipid coverage in these analyses, as the difference between the number of annotations is minimal. Further, the categories of lipids that were annotated in each replicate are comparable and provide additional support for the consistency of this method.

We also examined the reproducibility of lipid signal between replicates (Fig. 8C). For these analyses, the ratio of each common annotation was taken between Replicate 1 and Replicate 2, and then converted to a log_10_ scale (e.g., log_10_(Replicate 1: Replicate 2)). If the signal is equal between both replicates, the value for that annotation should be zero. Conversely, a positive or negative value, respectively, for this calculation means higher average abundance for Replicate 1 or Replicate 2. Overall, abundances between replicates were most reproducible in positive mode, specifically technical layers 1 and 5. While there appears to be a bias in detection in negative mode for Replicate 2, these values are still close to zero and could be suggestive of biological and/or analytical variability even though the mouse phenotypes are equivalent.

The results herein show support for the integration of AzC with a CA probe and top-hat IR-MALDESI for the purposes of 3D MSI as demonstrated on a well-characterized sample to this platform. Along with the advantages of more straightforward sample preparation and data analysis, this approach reduces the sampling bias across a ROI as a result of using a Gaussian laser profile and maintains the laser’s focus regardless of sample topography. The parameters optimized in this work successfully enabled high spatial resolution analyses in the *z*-dimension (i.e., 7 µm); however, other optical trains may be worth pursuing to maintain high spatial resolution in the *z*-dimension while also improving the *x*- and *y*-dimensions, as well. Furthermore, we recognize that the biological scope of this study was small, and a larger ROI across a tissue would be more informative to molecular changes when pursuing depth profiling experiments. Additionally, while sample material in this work was limited to maintain a consistent genotype for all specimens, future studies will be expanded to include more replicates to support strengthened conclusions regarding method reproducibility. Follow-on studies will be conducted on diverse sample types with more heterogeneous features (e.g., brain, whole-body models) to provide chemical elucidation of more complex model systems. Finally, incorporating automatic laser and CA probe calibration could improve reproducibility between samples and facilitate a faster, more robust source set-up to enable more comprehensive biological studies. Nevertheless, the technological advancements to the IR-MALDESI platform presented in this work enabled a foundation for insights into complex biomedical challenges that can be probed in detail by 3D MSI.

## Conclusions

This study demonstrates the first instance of leveraging top-hat IR-MALDESI with automatic *z*-correction for 3D MSI, where optimized experimental parameters for 3D MSI of skin successfully achieved a depth resolution of approximately 7 µm and minimized the sampling bias of a Gaussian laser beam. By this method, lipid heterogeneity was elucidated and chemically resolved the composition of epidermis and other dermal features in mouse skin. While this method shows reproducibility in lipid detection and abundance with repeated experiments, future studies will support the robustness of this approach for improved analytical variability. Ultimately, this work advances the field of MSI to present a new and technologically sound approach to 3D investigations for various biological questions.

## Supplementary Information

Below is the link to the electronic supplementary material.

Supplementary file1 (DOCX 2101 KB)
