# Supplementary material for: Three-dimensional mass spectrometry imaging (3D MSI): incorporating top-hat IR-MALDESI and automatic z-axis correction
Source: Anal Bioanal Chem. 2025 Feb 3;417(8):1649–61. doi: 10.1007/s00216-025-05755-w (PMC11876208; doi:10.1007/s00216-025-05755-w)
Supplement: Supplementary file 1 — Supplementary file1 (DOCX 2101 KB) [file 216_2025_5755_MOESM1_ESM.docx]

**Three-Dimensional Mass Spectrometry Imaging (3D MSI):**

**Incorporating Top-Hat IR-MALDESI and Automatic *z*-Axis Correction**

Alexandria L. Sohn^1^, John G. Witherspoon^2^, Robert C. Smart^2,3^, *David C. Muddiman^1^

*^1^FTMS Laboratory for Human Health Research, Department of Chemistry, ^2^Department of Biological Sciences, ^3^Center for Human Health and the Environment, North Carolina State University, Raleigh, NC 27695, USA*

**Supporting Information**

**Submitted to:** *Analytical and Bioanalytical Chemistry*

**Submitted:** January 17^th^, 2025

**Manuscript:** 3 Pages / 1 Supplemental Figure

**Keywords:** 3D, mass spectrometry imaging, lipids, automatic z-axis correction, top-hat

***Author for Correspondence**

David C. Muddiman, Ph.D.

FTMS Laboratory for Human Health Research

Department of Chemistry

North Carolina State University

Phone: 919-513-0084

Email: [dcmuddim@ncsu.edu](mailto:dcmuddim@ncsu.edu)

**Table of Contents**

**Figure S1** Top-hat spot size measurements and applied laser energies


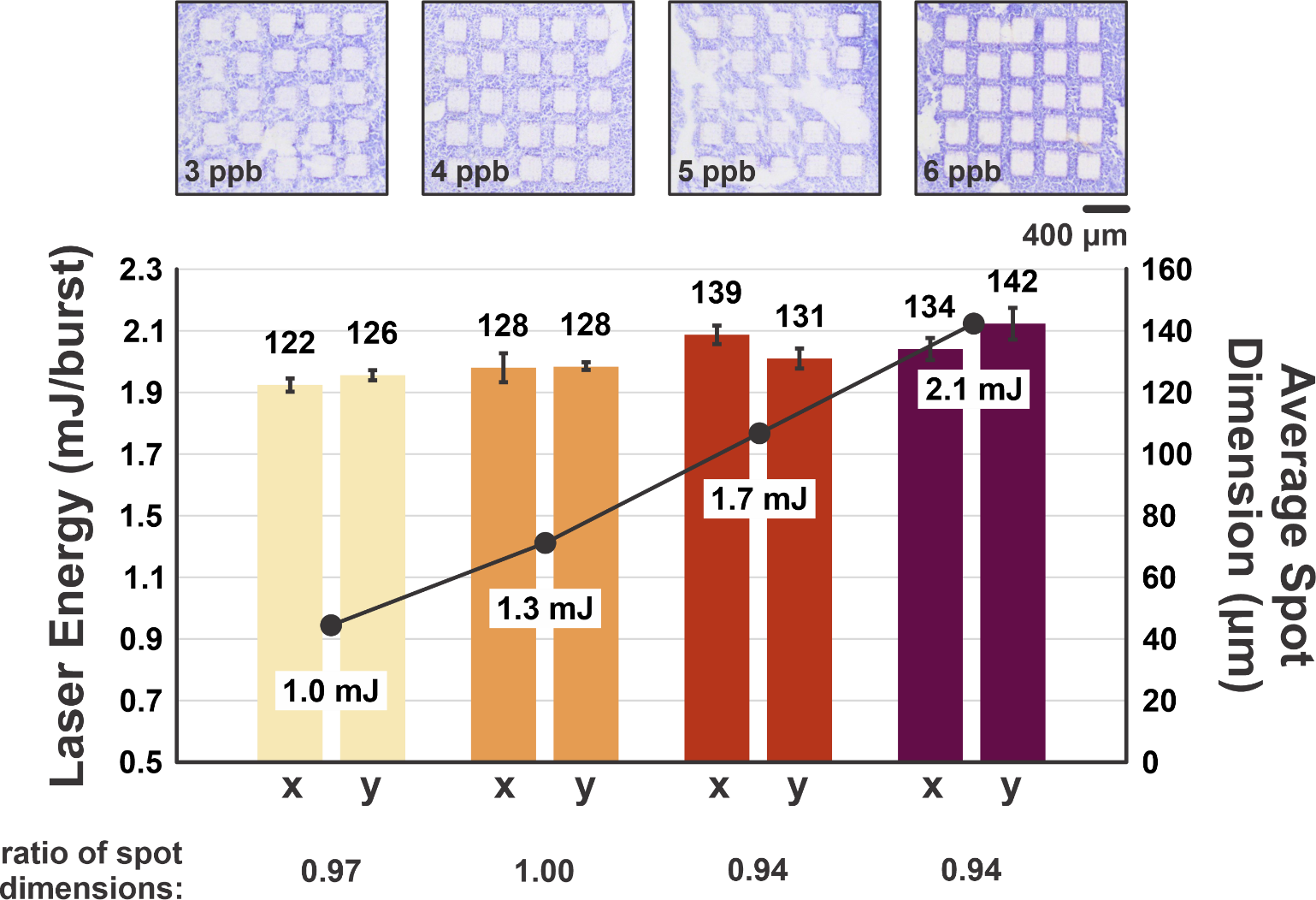


**Figure S1.** Different laser energies were considered for method development. At various pulses-per-burst (ppb), the laser energies varied from 1.0-2.1 mJ/burst (left y-axis) while maintaining a square spot shape on mouse liver tissue. The average spot dimensions for each laser energy are shown (n = 10) with the 95% confidence interval of the mean (right y-axis). The ratio of the x and y spot dimensions are reported below the x-axis.
